# Setting targets for HIV/AIDS—What lessons can be learned from other disease control programmes?

**DOI:** 10.1371/journal.pmed.1002735

**Published:** 2019-02-04

**Authors:** Tazeem Bhatia, Jamie Enoch, Mishal Khan, Sophie Mathewson, David Heymann, Richard Hayes, Osman Dar

**Affiliations:** 1 Public Health England, London, United Kingdom; 2 London School of Hygiene & Tropical Medicine, London, United Kingdom; 3 Chatham House Centre on Global Health Security, London, United Kingdom

## Abstract

In a Collection Review, Richard Hayes and colleagues discuss metrics for assessing progress in control of the HIV/AIDS epidemic in the context of prior disease control programmes.

**Summary points**

- Our analysis of experience from programmes targeting malaria, leprosy and TB shows the importance of drawing broadly on research and implementation expertise, and civil society more broadly, when setting targets for HIV control. The engagement of stakeholders from the highest burden settings, including affected populations, is crucial, to ensure that disease control efforts uphold human rights and tackle HIV-related stigma and discrimination.
- An appropriate balance is needed between ambitious, galvanising global targets that drive funding and political/public engagement, and targets that reflect the complexities and local epidemiological variations in disease profile. Ethical issues and unintended consequences need to be considered when setting targets—particularly around local effects and opportunity costs of having foregone other areas of disease control and public health. Intermediate and adaptable targets are needed that allow for course corrections to programmes.
- Overly burdensome reporting requirements for individual local programmes and countries should be avoided, as well as potential for overlapping and sometimes conflicting targets both within and across vertical disease programmes. Process targets should be distinguished from outcome targets, which should be measurable and based on high-quality data.
- Retention of expert healthcare worker skills and specialist services is vital, while moving towards integrated health systems if effective disease control programmes are to be maintained. Target development should seek areas of programme delivery where an opportunity to codevelop targets and integrate services exists. Global efforts to move to universal health coverage (UHC), for example, could be factored in when developing targets.
- Sustaining investment and continuing political interest in the end phase of any elimination or eradication strategy, once incidence and prevalence are low, are critical to achieve success. Equity- and access-based service delivery targets become increasingly important as the elimination strategy nears its end and should be factored into planning.
- Achieving disease elimination and/or eradication is only possible with sufficient investment in research to develop new prevention tools such as vaccines, point-of-care diagnostics, and treatments to counteract the effects of increasing drug resistance and the challenging latency period of diseases; public health infrastructure upgrades that address wider determinants of health; and health and surveillance systems that allow for equitable delivery and access to services.

## Introduction

Over the last four decades, efforts to address the global HIV pandemic have required multidisciplinary and multisectoral approaches adapted to different contexts [1]. The complex epidemiology of HIV and the breadth of scientific, societal, and political stakeholders involved in the global response have posed challenges in terms of coordination, harmonisation, and funding. Concerted advocacy and successive global strategies to prevent and control HIV have had a major influence on the global availability of political and financial support and on national response strategies [2]. However, the campaign against HIV highlights both the importance of globally agreed definitions and the challenges of developing a common understanding of overarching goals, targets, and measures of progress.

**Fig 1:**
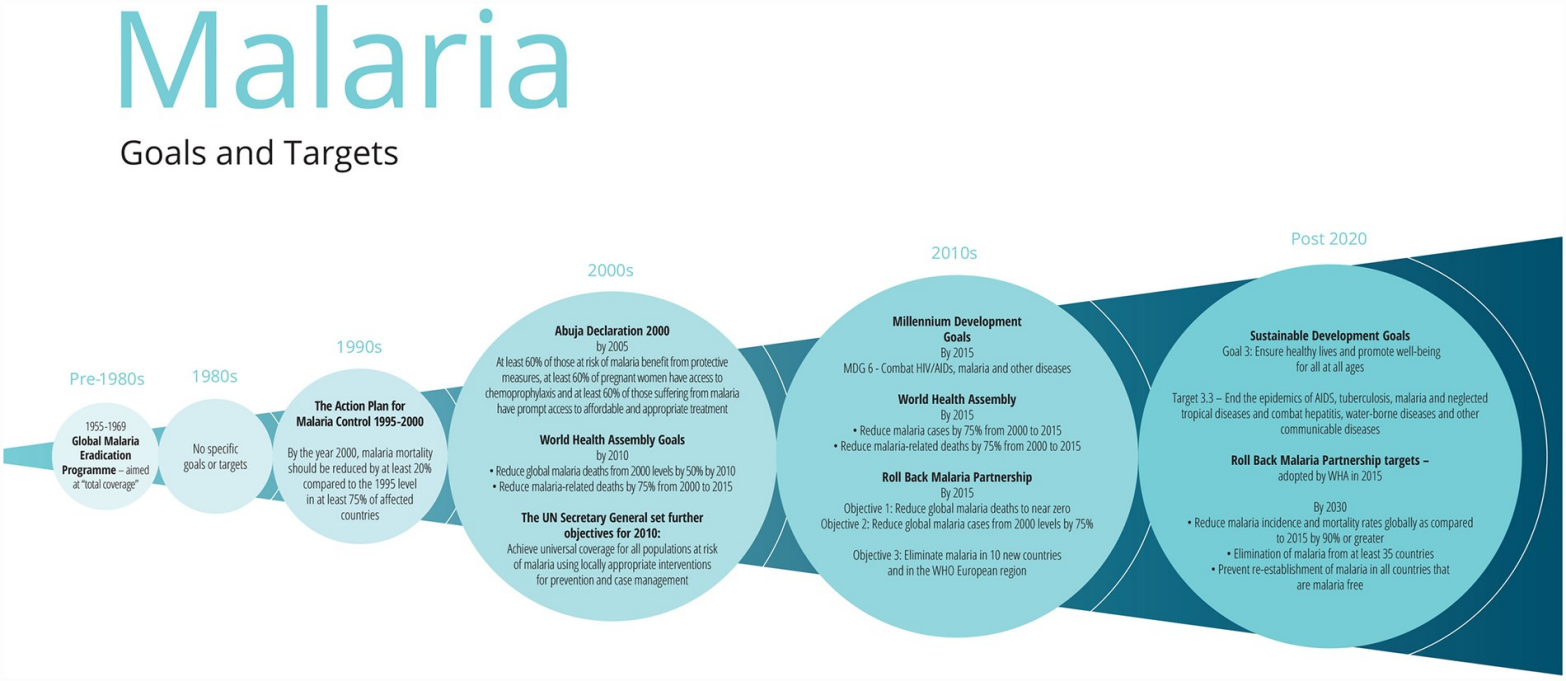
Major goals and targets for malaria. MDG, Millennium Development Goal; WHA, World Health Assembly.

**Fig 2:**
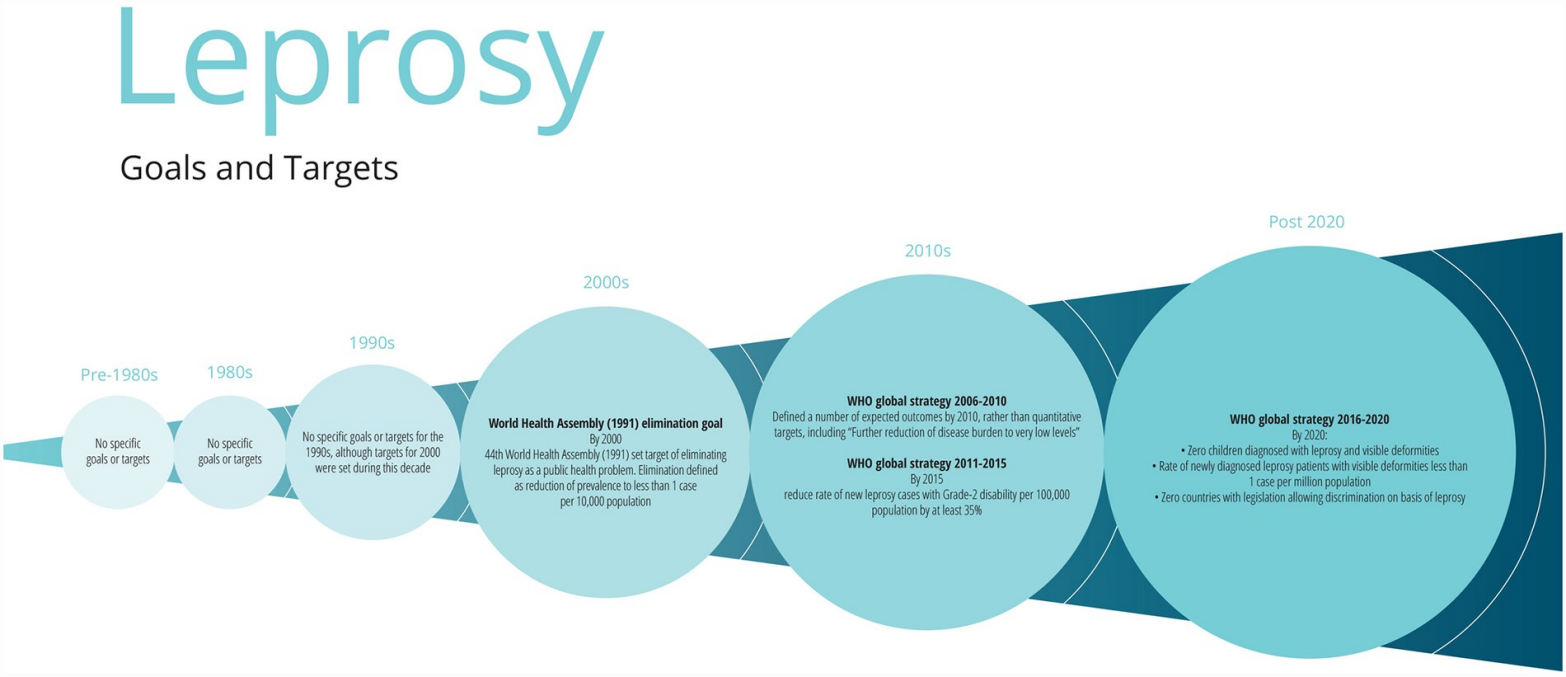
Major goals and targets for leprosy.

**Fig 3:**
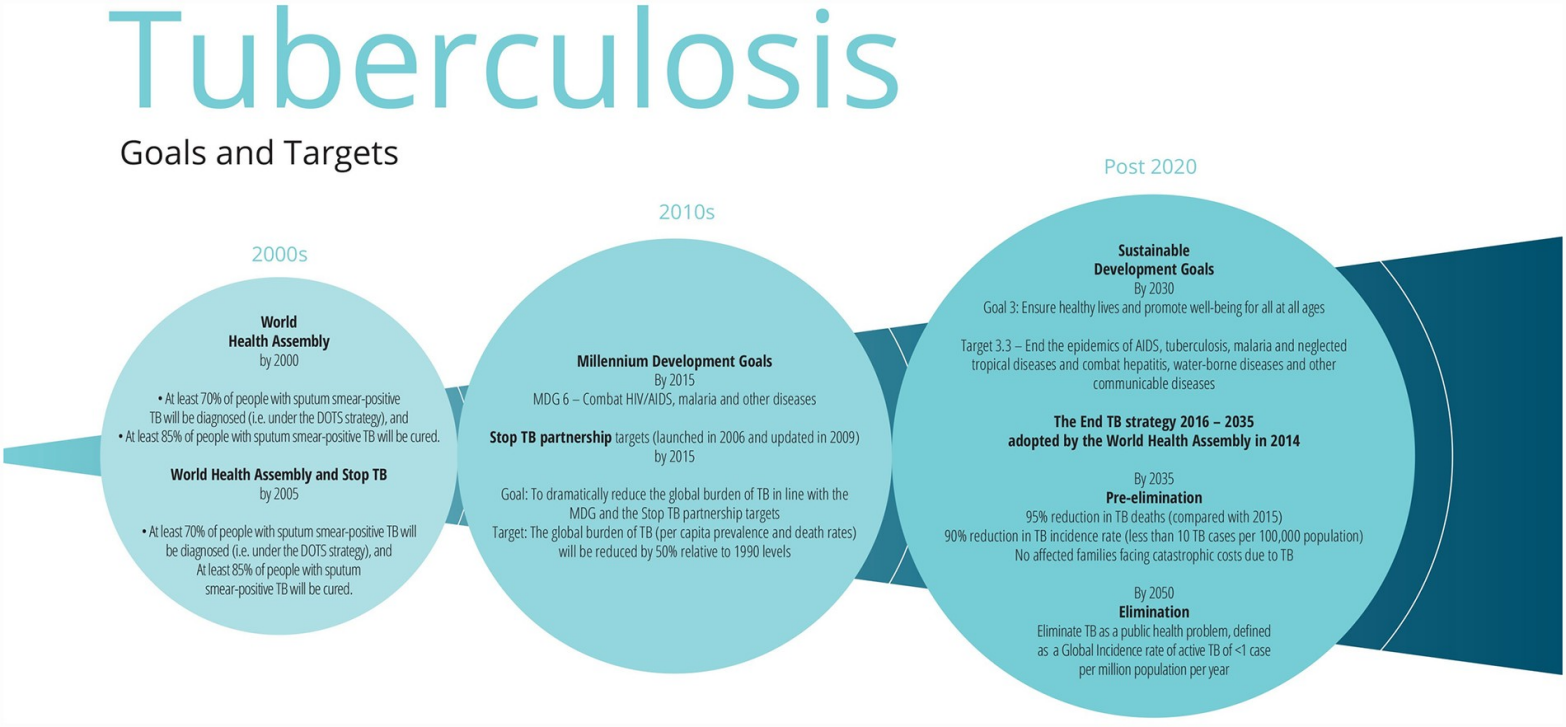
Major goals and targets for TB. DOTS, Directly Observed Treatment, Short Course; MDG, Millennium Development Goal; TB, tuberculosis.

**Fig 4:**
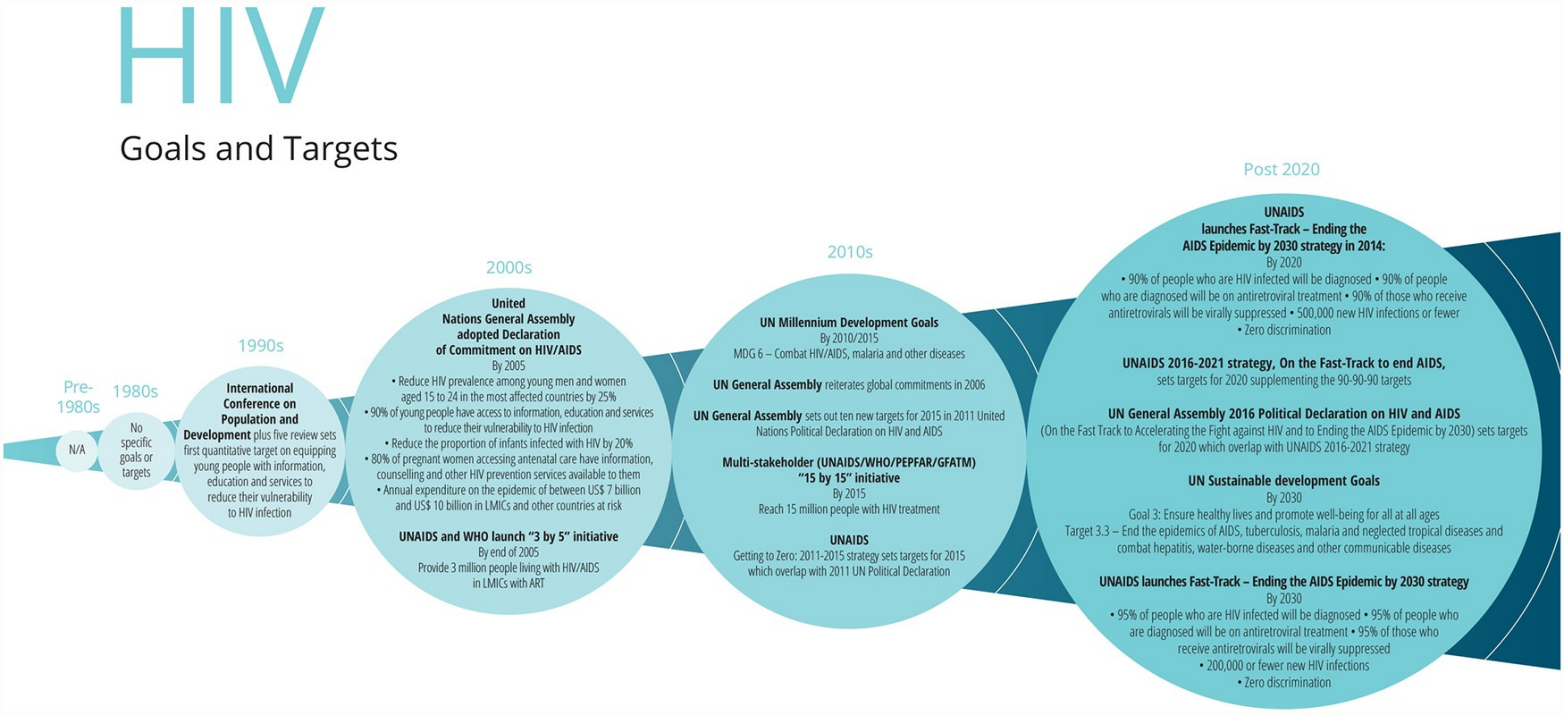
Major goals and targets for HIV/AIDS. ART, antiretroviral therapy; GFATM, Global Fund to Fight AIDS, Tuberculosis and Malaria; LMIC, low- or middle-income country; MDG, Millennium Development Goal; PEPFAR, US President’s Emergency Plan for AIDS Relief; UNAIDS, Joint United Nations Programme on HIV/AIDS.

As future global targets for the control of HIV are considered [3], we aim in this article to identify relevant lessons from control programmes for three other global infectious diseases—malaria, leprosy, and tuberculosis (TB). These were chosen because they have been the subject of international control efforts, with varying levels of success, and because they illustrate many of the problems faced by HIV control. These three programmes have faced challenges in reaching clear definitions of the concepts essential to epidemic control (Table 1), in sustaining political will and resources, and in meeting the needs of hard-to-reach subgroups. In the following sections, we briefly summarise epidemiological comparisons between these diseases and HIV (see Table 2) and then analyse the evolution of each disease control programme from the 1950s onwards, focusing on the stated global control strategy, specific targets, and major global events or initiatives (see S1 Table and Figs 1–4). Finally, we discuss how experience from these programmes may inform the setting of future goals and targets for HIV.

**Table 1:** Essential terms and concepts for defining goals and targets to limit infectious disease epidemics [4].

**Control**	Nonspecific term for reduction of disease incidence, prevalence, morbidity, and/or mortality to a locally acceptable level as a result of deliberate efforts; continued intervention measures are required to maintain the reduction.
**Elimination of transmission**	A reduction to zero of the transmission of infection caused by a specific pathogen in a defined geographical area, with minimal risk of reintroduction, as a result of deliberate efforts; continued actions to prevent re-establishment of transmission may be required.
**Verification**	The process of documenting elimination of transmission.
**Elimination as a public health problem**	Defined by achievement of measurable global targets set in relation to a specific disease. When reached, continued actions are required to maintain the targets and/or to advance the interruption of transmission.
**Validation**	The process of documenting elimination as a public health problem.
**Eradication**	The permanent reduction to zero transmission of a specific pathogen as a result of deliberate efforts, with no more risk of reintroduction.
**Certification**	The process of documenting eradication.
**Extinction**	Eradication of a specific pathogen so that it no longer exists in nature or the laboratory.

**Table 2:** Epidemiological comparison of the four diseases considered.

Features	Malaria	TB	Leprosy	HIV/AIDS
**Transmission**	Caused by the *Plasmodium* parasite and spread to people through the bites of infected female *Anopheles* mosquitoes. There are five parasite species (including *P*. *falciparum* and *P*. *vivax*) that cause malaria in humans [5]. There is no animal reservoir in nature.	Caused by a bacterium (*Mycobacterium tuberculosis*) and most often affects the lungs. Spread from persons with active infection through droplets in the air. Approximately one third of the world’s population has latent TB (long-term asymptomatic infection), but they have only a 10% lifetime risk of becoming sick with TB. If not treated, each person with active TB infects on average 10 to 15 people each year [6]. The proportion of human cases of TB caused by *M*. *bovis* (bovine TB) is estimated at <5% [7].	Caused by a bacterium (*M*. *leprae*). Transmission is favoured by close contact. May be transmitted from nasal mucosa, possibly through respiratory secretions, but the exact mechanism is not clearly understood. Over 85% of clinical cases are noninfectious. Evidence suggests infectiousness is lost in most instances within a few days of beginning MDT [8]. Zoonotic transmission of leprosy from armadillos to humans has been recorded in the Southern United States and parts of South America [9].	Caused by the human immunodeficiency virus; left untreated (2 to 15 years postinfection), acquired immunodeficiency syndrome (AIDS) develops. HIV is transmitted person to person by unprotected sexual intercourse; use of HIV-contaminated injecting and skin-piercing equipment; vertically from mother to infant during pregnancy, delivery, or breastfeeding; or transfusion of infected blood or its components [10].
**Who is at risk?**	Those at highest risk of severe disease are those with the lowest immunity: infants and young children, pregnant women and patients with HIV/AIDS, nonimmune migrants, mobile populations, and travellers. Seventy percent of malaria deaths occur in children under 5 years [5].	Active TB mostly affects adults in their productive years. People who are infected with HIV are 20 to 30 times more likely to develop active TB. The risk of active TB is also greater in persons suffering from malnutrition or diabetes, and smokers [6].	Persons at highest risk live in endemic areas in close contact with multibacillary cases. Genetic factors play a part in determining the risk of disease [11]. Leprosy reactions may be masked in patients with advanced HIV disease. Children under 14, as well as older adults, may be at particular risk of leprosy infection [12].	Heterosexual sexual transmission is the predominant mode of HIV transmission in the sub-Saharan Africa and South-East Asia regions, with young women particularly vulnerable. Many epidemics are occurring among high-risk groups, including sex workers and men who have sex with men. Injecting drug use is a major mode of transmission in Eastern Europe and Central Asia. Without preventive interventions, the likelihood of transmission from infected mothers to their children is 15%–45% [13].
**Disease burden**	In 2017, ongoing malaria transmission was present in 90 countries and areas. There were an estimated 219 million cases of malaria, 435,000 deaths, and the incidence rate of malaria was estimated at 63 cases per 1,000 population at risk. A total of 92% of malaria cases are in Africa and 93% of malaria deaths. *P*. *falciparum* is the most prevalent malaria parasite in sub-Saharan Africa, accounting for 99% of estimated malaria cases [5].	Over 95% of cases and deaths are in LMICs. In 2016, 45% of new cases occurred in Asia, and 25% in Africa. A total of 74% of people coinfected with TB-HIV in 2016 live in Africa [6]. Globally in 2016, there were an estimated 1.7 billion people infected with TB and an estimated 10.4 million incident cases of TB (range, 8.8 million to 12.2 million), equivalent to 140 cases per 100,000 population [14].	In 2017, the estimated global prevalence of leprosy was 0.25 per 10,000 population, and the rate of detection of new cases was 2.77 per 100,000 population [15]. In 2015, India reported 127,326 newly diagnosed cases (60% of those reported globally); Brazil reported 26,395 (13% of those reported globally); and Indonesia reported 17,202 (8% of those reported globally) [16]. The majority of countries with a high number of newly diagnosed cases are located in the Africa and South-East Asia WHO regions.	Approximately 36.9 million people were living with HIV at the end of 2017 globally, and 1.8 million people became newly infected with HIV in 2017. Sub-Saharan Africa is the most affected region, with 25.7 million people living with HIV in 2017. The WHO Africa region accounts for over two thirds of the global total of new HIV infections each year. Current estimates suggest that 75% of people living with HIV know their status [17].
**Prevention**	Vector control by use of insecticide-treated mosquito nets and indoor residual spraying. Intermittent preventative treatment for pregnant women and infants in areas of moderate to high malaria transmission in Africa. Seasonal malaria chemoprevention in children under the age of 5 years in areas of high seasonal transmission in the Sahel, Africa [5].	Early diagnosis and treatment to stop transmission, chemoprophylaxis for latent TB in young children and those coinfected with HIV or immunocompromised, and BCG vaccine [14].	Early detection and prompt MDT of cases, and evaluation and treatment of infected household contacts. Early detection and treatment with MDT has prevented about 4 million people from becoming disabled [8].	Safer sexual behaviour, including male and female condom use; testing and counselling for HIV and STIs; medical male circumcision; ARV drug use for prevention (pre-exposure prophylaxis) and treatment as prevention; harm reduction for injecting drug users; elimination of mother-to-child transmission [18].
**Diagnosis**	Parasite-based diagnostic testing—can either be by microscopy or rapid diagnostic test—for which results can be available within 30 minutes [5].	Most countries still rely on sputum smear microscopy; however, microscopy detects only half the number of TB cases and cannot detect drug resistance. Since 2010, the rapid test Xpert MTB/RIF has become more available. Diagnosis can be made within 2 hours, requires less technical expertise, and can detect resistant strains [6].	Clinical diagnosis is based on complete skin examination, involving identification of skin lesions, peripheral nerve involvement, or motor weakness and sensory loss. Laboratory diagnosis is through identification of acid-fast bacilli in slit skin smears or by full thickness skin biopsy. In practice, laboratory studies are not essential for the diagnosis of leprosy, although confirmation by skin biopsy is recommended [19].	Serological tests (e.g., rapid diagnostic tests or enzyme immunoassays) can detect the presence or absence of antibodies to HIV-1/2 and/or the HIV p24 antigen [20]. HIV self-testing does not provide a definitive diagnosis but is an initial test that requires further testing by a health worker using a nationally validated testing algorithm [21].
**Treatment**	Malaria is curable. For *P*. *falciparum*, malaria ACT is recommended [5]. However, counterfeit antimalarials are an increasing global problem [22].	The great majority of TB cases can be cured by four antimicrobial drugs taken properly over 6 months. However, MDR TB and extensively drug-resistant TB do not respond as well to second-line treatments and can require 2 or more years of treatment. In 2016, 35 Asian and African countries saw the introduction of new second-line drugs that have shortened the length of MDR TB regimens [6].	MDT—combined chemotherapy with rifampicin, dapsone, and clofazimine—is available free of charge to most countries from WHO or through national health programmes. The number of skin lesions is used to guide treatment. The standard WHO regimens are (a) a 12-month oral course of MDT for adults with more than five skin lesions and (b) a 6-month oral course of rifampicin and dapsone for adults with two to five skin lesions [23].	Combination ART consisting of three or more ARV drugs can control the virus by lowering viral load and helping prevent onward transmission. New WHO guidelines in 2016 recommended provision of lifelong ART to all children, adolescents, and adults, including all pregnant and breastfeeding women living with HIV, regardless of CD4 cell count [24].
**Resistance**	Vector control is dependent on the use of pyrethroids, the only class of insecticides recommended for ITNs or LLINs. Mosquito resistance to pyrethroids has emerged, but there is believed to be no decreased efficacy of LLINs. Rotational use of different classes of insecticides for indoor spraying is one approach to managing resistance [5]. Resistance to antimalarial medicines is also a recurring problem, with chloroquine and sulfadoxine-pyrimethamine (SP) developing resistance in the 1950s and 1960s. Recently, parasite resistance to artemisinin has been detected in five countries of the Greater Mekong subregion [5].	Resistant TB strains have developed through the use of incorrect prescriptions, poor quality drugs, and patients stopping treatment prematurely. MDR TB is resistance to two of the most powerful first-line drugs, rifampicin and isoniazid. In 2016, there were 490,000 people with MDR TB, approximately half of whom lived in China, India, or the Russian Federation, but only 54% were successfully treated. In 2016, about 6.2% of MDR TB cases had XDR TB, which means resistant to second-line drugs, and only 30% were successfully treated [6].	In the 1960s, *M*. *leprae* started to develop resistance to dapsone, the world’s only known anti-leprosy drug at that time. Today, there are ‘a few isolated reports of rifampicin-resistant leprosy, mainly from areas where rifampicin was given as monotherapy, either alone or in combination with dapsone, to dapsone-resistant patients’ [25]. Resistance to rifamipicin, the most important component of MDT, appears to be associated with noncompliance with dapsone or clofazimine regimens.	HIV drug resistance rapidly appears if only one or two ARV drugs are used, if treatment adherence is poor, or if there are interruptions in treatment. The rollout of ART has been accompanied by increases in resistance at the population level; research has shown that in the first 10 years (2001–2011) of mass treatment rollout, non-nucleoside reverse transcriptase inhibitors resistance increased by 36% per year in East Africa and by 23% in Southern Africa [26]. A recent WHO report found that urgent attention is needed to decrease levels of loss to follow-up, support retention, maximise adherence, and prevent drug stock outs [27].
**Vaccine development**	More than 30 *P*. *falciparum* malaria vaccine candidates are at advanced preclinical or clinical stages of evaluation, but only the RTS,S/AS01 vaccine has completed Phase 3 trials and passed a positive regulatory assessment. In November 2016, WHO announced that the RTS,S vaccine would be rolled out in pilot projects in three countries in sub-Saharan Africa [5]. Potential shortcomings of the vaccine are that it is partially effective and requires three primary doses followed by a booster [28]. Of the trial participants given a four-dose schedule starting at 5–17 months of age, vaccine efficacy against severe malaria was 31.5% (95% CI 9.3–48.3) over about 4 years of follow-up [29].	BCG vaccine does prevent infection and is partially effective in preventing miliary TB in young children. A new vaccine that can prevent infection is key to addressing the reservoir of infection required to achieve the End TB strategy. There are 16 different TB vaccine candidates, but none have passed Phase II trials yet [30].	BCG vaccine has a protective effect against leprosy. One meta-analysis suggested an overall protective effect of 26% based on seven experimental studies [31]. Trials are underway in India to investigate protection provided by a vaccine based on *M*. *indicus pranii* [32].	HIV vaccine development is complicated by the extreme variability of the virus and, in particular, its envelope protein at both the individual and population level. A large multiyear clinical trial (HVTN 702) of a new vaccine is currently underway in South Africa [33].

Abbreviations: ACT, artemisinin-based combination therapy; ART, antiretroviral therapy; ARV, antiretroviral; BCG, bacille Calmette-Guérin vaccine; HVTN, HIV Vaccine Trials Network; ITN, insecticide-treated bednet; LLIN, long-lasting insecticide-treated bednet; LMIC, low- or middle-income country; MDR TB, multidrug-resistant TB; MDT, multidrug therapy; SP, sulphadoxine-pyrimethamine; STI, sexually transmitted infection; TB, tuberculosis; XDR TB, extensively drug-resistant tuberculosis.

## Malaria

In 1955, the World Health Assembly (WHA) adopted a Global Malaria Eradication Campaign (GMEP) involving indoor spraying with dichlorodiphenyltrichloroethane (DDT) and treatment of malaria [34]. Eradication was defined as the ‘global extinction’ of the parasite [35].

The GMEP succeeded in eliminating malaria from many parts of the world [34], but in sub-Saharan Africa, where mortality is highest, eradication was deemed technically unfeasible and was finally abandoned in 1969. In retrospect, it was clear that the humanitarian appeal of the campaign and subsequent urgency had led to an oversimplification and standardisation of the programme. In the ‘consolidation phase’, the improvements in malaria control could not be maintained without well-developed public health infrastructure. The continued high expenditure in an environment of greatly reduced transmission and disease incidence was difficult to defend. And finally, increasing resistance both of vectors to DDT and the parasites to chloroquine sealed the GMEP’s fate [34]. These lessons are relevant to attempts to end any disease, including HIV/AIDS.

The following two decades saw a resurgence of the disease due to de-skilled and under-resourced programmes, compounded by political instability from multiple conflicts and postcolonial turmoil. The Roll Back Malaria (RBM) initiative of the 1990s was the first sign of renewed international interest [36].

In October 2007, the Bill & Melinda Gates Foundation, endorsed by WHO, announced a renewed ambition to eradicate malaria. However, most technical experts agree that malaria elimination and subsequent eradication cannot be achieved with existing tools but will require the development of new tools and approaches [37,38].

Malaria elimination, defined as a state in which interventions have interrupted endemic transmission within a geographic area with minimal risk of re-establishment [35], may be realistic for Asia, southern America, and parts of Africa. South Africa is aiming to eliminate malaria before 2020 [39]. Elimination is a particularly attractive target for combating drug resistance in Southeast Asia. But will this process be time limited or will sustained suppression through control efforts be required indefinitely [35]? Socioeconomic and environmental development in Europe and North America allowed for the relaxation of control measures there. The development of a highly effective vaccine that can interrupt transmission and decrease the risk of transmission from mosquitoes, or gene editing of female mosquitoes so that they can carry only male-producing eggs may be important parts of a solution [40].

As a result of scaling up malaria control efforts, between 2000 and 2015, the world saw an estimated 37% reduction in incidence and a 60% decline in mortality rates [41], thus realising Millennium Development Goal (MDG) 6. However, the WHA target to reduce the global burden of malaria by 75% by 2015 [42] and the RBM target to reduce deaths to near zero by the end of 2015 have not been attained [43]. In 2017, there were 219 million cases of malaria globally, 92% of them in Africa, of which 99% were *Plasmodium falciparum* where the incidence rate in 2016 was 206 cases per 1,000 population at risk [5].

The recent Global Technical Strategy for malaria 2016–2030 [44] advocates a combination of control measures for highly endemic regions (reducing mortality by 90% and incidence by 90% compared with 2015), investment in malaria elimination in 35 countries with low malaria incidence [45], preventing re-establishment in malaria free countries, and research into developing novel interventions. Given the shortfall in financial resources (in 2017, US$3.1 billion were invested, half the minimum US$6.5 billion estimated to be required) [5], investment in shrinking the malaria map should not be at the expense of funding for countries with the highest burden of disease.

## Leprosy

Both leprosy and HIV/AIDS are chronic rather than acute infectious diseases, requiring long-term management and treatment, and disproportionately affecting marginalised groups. However, leprosy differs from HIV in that multidrug therapy (MDT) cures leprosy infection and can be discontinued, while HIV requires lifelong treatment to suppress, not cure, infection.

A surge in funding for MDT rollout, shortened treatment regimens, and changing case definitions of leprosy led to a considerable decrease in recorded leprosy prevalence by the end of the 1980s [46]. Amid global progress and optimism, the WHA passed a resolution in 1991 seeking to ‘eliminate leprosy as a public health problem by 2000’, defining elimination as reduction of prevalence to less than 1 in 10,000 globally. The 1/10,000 target was chosen arbitrarily, with limited consultation [47]; it was thought that attaining this prevalence would eventually interrupt leprosy transmission, but it was not supported by evidence (e.g., from modelling) [48].

At the global level, the prevalence target of 1/10,000 was achieved by the end of 2000, with all but six countries reaching the target nationally by 2005. (Success in achieving the target was contingent on the use of the whole world population—including populations with very few leprosy cases, e.g., in Northern Europe—as the denominator.) However, particularly in countries with a significant disease burden such as India and Brazil, continuing high numbers of new cases detected indicated that MDT had not interrupted transmission as anticipated [49]. Since 2005, declines in both prevalence and incidence rates have largely stalled [50], and many countries with a national prevalence below 1/10,000 continue to have high incidence subnationally [51]. Arguably, attaining the global target has represented an advance in leprosy control rather than elimination [52]. In 2017, the estimated global prevalence of leprosy was 0.25 per 10,000 population, and the rate of detection of new cases was 2.77 per 100,000 population [15].

Leprosy elimination efforts have been complicated by the disease’s long latent period (of up to 20 years), the lack of a single diagnostic tool enabling early detection, and the complex and varied clinical presentation. From an operational perspective, recent declines in case detection and case identification activities in high-endemic settings have negatively affected diagnosis and treatment coverage [53]. Indeed, there is evidence that pressure to achieve the elimination target at the national level may have led to less active case finding, diagnosis, and reporting, thus artificially resulting in lower prevalence figures [54].

A 2003 independent evaluation of the Global Alliance for the Elimination of Leprosy recommended a leprosy control approach focused on rehabilitation and preventing nerve damage, rather than elimination [55]. WHO officially abandoned the elimination target of 1/10,000 in 2007, and newer targets in WHO’s five-yearly plans have shifted towards prevention of secondary disability, with targets for the reduction of grade 2 disabilities (G2D, defined as visible deformity or damage present in the hands and feet, or severe visual impairment). However, despite the 2011–2015 WHO plan’s target of reducing the rate of G2D by 35% [56], there was no decrease in G2D between 2010 and 2013 [50].

The 2016–2020 strategy includes targets around reducing G2D and discrimination, underpinned by 23 performance indicators, six guiding principles, and three pillars [57]. It is instructive for HIV control to compare the complexity of the current leprosy control targets with the simple elimination target chosen in 1991. The advocacy and resources behind the 1/10,000 prevalence target put leprosy firmly on the global health agenda and helped detect and cure many cases. The no-cost delivery of MDT to endemic countries meant that, at the global level, the elimination programme was highly equitable (although some individuals had poor outcomes) [58]. However, the perception that elimination was imminent, even as transmission continued, resulted in reduced funding for leprosy programmes in the 2000s and the loss of specialist leprosy services, as diagnosis and management of leprosy became integrated into peripheral health services [59]. The elimination target also became politically charged, leading to tensions between civil society organisations and global or national leprosy programmes [55]. Furthermore, elimination rhetoric may have reduced scientific interest in leprosy, despite significant evidence gaps [50]. For example, the *International Journal of Leprosy* ceased publication in 2005 [60], while research and development on vaccines has largely stalled (although a new clinical trial of a candidate vaccine was launched in India in 2017) [61].

In terms of implications for HIV control, the example of leprosy highlights both the benefits and hazards of setting strong, high-level targets. While there was significant progress in expanding access to treatment, the initiative did not recognise the complexity of leprosy elimination and did not take into account the importance of long-term disability of individuals who had ceased to be counted as cases. Leprosy demonstrates the misalignment between achieving a high-level target and implementation, in which efforts often did more to control than to end leprosy.

## TB

Following the Second World War, industrialised countries witnessed rapid declines in TB incidence of approximately 10% per year [62] associated with socioeconomic development, including reductions in overcrowding and improved living conditions, nutrition, and hygiene [63]. Effective TB control was further aided by the advent of chemotherapy, universal access to healthcare in many countries, and TB-specific vertical programmes.

By the early 1960s it had become clear that a vertical programme approach was too costly for low-income countries. From the mid-1960s there was a move to integrate TB service delivery into general health services, with the hope of increasing coverage and reducing costs [63].

The early 1990s witnessed a sharp rise in TB notifications linked to the advent of the HIV epidemic and dissolution of the former Union of Soviet Socialist Republics’ (USSR) structures. In 1993, WHO declared a ‘global TB emergency’ [64], and targets were set for the turn of the millennium that aimed at reducing TB incidence by 5%–10% annually [65]. A new Directly Observed Treatment, Short Course (DOTS) strategy was launched, focussing primarily on increasing cure rates and improving case detection. A vertical DOTS-based strategy was widely promoted, with specialist managerial functions implemented at central, regional, and district levels and delivery carried out through primary healthcare infrastructure [66]. Although drug-resistant TB was recognised at this point, the DOTS strategy assumed that increasing adherence and cure rates would prevent the spread of multidrug-resistant TB (MDR TB), and access to MDR TB treatment was limited.

The targets for 2000 were met in 2005 due to improvements in living conditions and DOTS in China, India, and Indonesia, the countries with the highest burden of disease then and now [14], but many countries did not meet them. The Global Plan to Stop TB, 2006–2015 set bold new targets to halve the prevalence and death rates of TB by 2015 (as compared with the estimated prevalence in 1990).

By 2015, with active TB incidence falling by an average of 1.5% per year since 2000, the MDG to ‘halt and reverse TB incidence’ was met globally [67]. Globally, TB mortality has fallen by 47% between 1990 and 2015, and the prevalence was almost half that of the 1990 estimate [67]. However, there is considerable variation in outcomes between countries, and with the growth of populations, the absolute number of new TB infections globally has grown, hence the current approach to target high-burden countries, similar to the US President’s Emergency Plan for AIDS Relief (PEPFAR) approach to HIV [68].

In 2014, the WHA approved the WHO End TB strategy, 2016–2035, which sought to reduce annual incidence of active TB to less than 10 cases per 100,000 population by 2035. This would mean that, of the 8.5 billion people expected to be alive in 2035, the number of new cases of active TB would need to be fewer than 900,000 [69], as compared with the estimated 10.4 million new TB cases in 2016, equivalent to 140 cases per 100,000 population [14]. The decline in active TB incidence has never been more than 1%–2% per year at the global level [70]. To meet this target, the strategy ambitiously assumes that the incidence rate falls at 10% annually between 2015 and 2025 through the optimisation of current tools and significant progress in achieving the Sustainable Development Goals (SDGs), and then declines further at an average of 17% annually with the advent of new technologies including a vaccine, new drug treatments for active and latent disease, and point-of-care diagnostics [71]. Modelling studies suggest that achieving the SDGs would have the most significant impact on the incidence of active TB, but most of our efforts still focus on improving drugs and technologies [72]. Key challenges include preventing, diagnosing, and treating MDR TB. In 2016, approximately 22% of MDR TB patients were enrolled in second-line treatment, while treatment success is only 54% globally. But also important is the large, global reservoir of latent TB infection. Even if transmission were completely interrupted from now onwards, reactivation and relapse would still generate more than 10 active cases per 100,000 in 2050 [69]. Active TB incidence could be brought down quickly with the discovery of a vaccine to prevent infection and a postinfection vaccine that could neutralise the reservoir of latent infection [69].

Achieving global TB control requires high-incidence countries to have systems and strategies to be able to accurately diagnose and deliver treatment early, universal healthcare coverage, and social protection to achieve high cure rates of active TB and MDR TB, coupled with the necessary social and economic development to sustain achievements. In short, the End TB Strategy elimination targets cannot be achieved without both rapid and substantial progress towards the SDGs [73,74], and the development of a simple and effective mechanism for managing latent infection.

## HIV

Early in the HIV/AIDS epidemic, there were limited targets at the global level. These have since developed into increasingly numerous, complex target frameworks. This section briefly summarises different HIV targets at the global level, omitting the many specific national- and community-level targets.

High-profile global HIV targets were first defined in the 2000 MDGs. The MDGs did not specify quantitative targets, stating an aim to ‘halt and reverse’ the epidemic, without defining this precisely [75]. The Declaration of Commitment on HIV/AIDS of 2001 set out more detailed time-bound goals and targets [76], with an emphasis on transparency, accountability, and ongoing reporting that owes much to concerted, determined civil society activism [77]. Progress against the 2001 Declaration’s targets was systematically reported in 2006 [78], demonstrating that only the target on increasing HIV/AIDS funding for low- and middle-income countries (LMICs) had been achieved, while there were significant gaps in progress towards the prevention and epidemiological targets (Table 3).

**Table 3:** Targets set in the UNGASS 2001 declaration of commitment on HIV/AIDS.

Global target set in 2001 Declaration	Indicator (cited in 2006 report)	Global result reported in 2006
Reduce by 2005 HIV prevalence among young men and women aged 15 to 24 in the most affected countries by 25% (Paragraph 47)	Percentage of young men and women aged 15–24 who are infected with HIV	Inconclusive (at the global level). 2006 report gives global prevalence measures for this age cohort: ‘Women: 4.1% (Measure of uncertainty: 3.2%–5.1%) Men: 1.6% (Measure of uncertainty: 1.2%–2.0%)’ but states, ‘No comparable global data on this age cohort is available from 2001. Progress towards target can only be measured in individual countries.’
Ninety percent have access to information, education, and services to reduce their vulnerability to HIV infection (Paragraph 53)	Percentage of youth aged 15–24 who correctly identify ways of preventing HIV transmission	(Males) 33% (Country range: 7%–50% coverage) (Females) 20% (Country range: 8%–44% coverage)
By 2005, reduce the proportion of infants infected with HIV by 20% (Paragraph 54)	Estimated percentage of infants born to mothers infected with HIV who are infected in 2005	26% (in countries with generalised epidemics). ‘There has been an estimated 10% reduction in HIV transmission between 2001 and 2005.’
Eighty percent of pregnant women accessing antenatal care have information, counselling, and other HIV prevention services available to them, increasing the availability of and providing access for women and babies infected with HIV to effective treatment to reduce mother-to-child transmission of HIV (Paragraph 54)	Percentage of HIV-positive pregnant women receiving ARV prophylaxis	9% (Country range: 1%–59% coverage)
Annual expenditure on the epidemic of between US$7 billion and US$10 billion in LMICs and countries experiencing, or at risk of experiencing, rapid expansion of HIV/AIDS (Paragraph 80)	Total annual expenditure	US$8,297,000,000 Estimated range: $US7.4 billion–US$8.5 billion

Abbreviations: ARV, antiretroviral; LMIC, low- or middle-income country; UNGASS, UN General Assembly Special Session.

Thanks in large part to pressure from activists to expand access to antiretroviral therapy (ART), quantitative targets for ART coverage began with WHO’s commitment to enrol 3 million people on treatment by 2005. Later, the Joint United Nations Programme on HIV/AIDS’s (UNAIDS’s) 15 by (20)15 initiative aimed to scale up and sustain ART coverage for 15 million people, a target reached ahead of schedule in March 2015; this was heralded as fulfilment of the MDG to ‘halt and reverse’ the epidemic [79].

UNAIDS’ 2011–2015 strategy set ambitious targets beneath a broader vision statement of the Three Zeros—zero new HIV infections, zero AIDS-related deaths, and zero discrimination—which continues to frame the AIDS response [80]. The year 2014 saw the development of the Fast Track strategy [81], setting out 10 targets, one of which was the ‘90-90-90’ target for 2020, which remains a significant marker of progress for countries. The 90-90-90 target aims for 90% of persons living with HIV/AIDS (PLHIV) to be diagnosed, 90% of diagnosed people to be on ART, and 90% of people on ART to have a fully suppressed viral load. Globally, UNAIDS estimates that 75% of PLHIV knew their HIV status at the end of 2017. Their estimates also suggest that 79% of diagnosed PLHIV were accessing treatment, and 81% of PLHIV in treatment were virally suppressed [82]. The Fast-Track strategy has a particular focus on the 30 countries with the highest number of new infections, with each country defining its own approach and 2020 milestones alongside the global targets.

The relative granularity of this approach contrasts with the more high-level 2015 SDG on HIV (3.3): ‘By 2030, end the epidemics of AIDS…’, with the target indicator (3.3.1): ‘Number of new HIV infections per 1,000 uninfected population, by sex, age and key populations’ [83,84]. Like the MDG target, the epidemiological measure defining the epidemic’s end is not explicitly stated.

Targets set in the UNAIDS 2016–2021 strategy [85] and the 2016 Political Declaration on Ending AIDS [86] demonstrate the difficulty of evaluating progress against complex targets when high-quality data are not available. For example the target in the 2016 Declaration, ‘Ensure that at least 30% of all service delivery is community-led by 2020’ (Fast Track Commitment 7 [87]) is challenging to monitor. The 2017 report on the 2016 Declaration by the UN Secretary General [88] captures examples of good practice in community-led service delivery but does not include quantitative data on global progress towards this target.

Several targets today are sensitive to the significant variation in the burden of HIV among different subpopulations, recognising inequalities in vulnerability to HIV. For example, the 2016 Political Declaration includes a commitment to reduce the number of new HIV infections among adolescent girls and young women to below 100,000 per year (Commitment 5). This demonstrates that targets are seeking to respond to structural drivers of the epidemic, even if the available data suggest progress is too slow to address these; for example, in 2016, there were 360,000 new HIV infections among adolescent girls and young women, only a 17% decline since 2010 [89].

As discussed above, many process targets relating to funding provision, ART coverage, and elimination of vertical HIV transmission have been achieved in several countries. However, there has been more limited progress towards impact targets, for example, around the reduction of incidence or expanding access to combination prevention among key populations. This highlights the inherent ambition of ‘Ending AIDS as a public threat’, given current rates of progress towards the constituent targets. Paradoxically, there is a risk that insufficiently nuanced rhetoric implying the end is in sight may deprioritise HIV, even while data illustrate that renewed financing and political will are critical to achieving progress towards an aspirational ‘end of AIDS’.

## Discussion

Several important lessons emerge from our analysis that should be considered in developing future goals and targets for HIV control.

### Engagement of stakeholders as well as multidisciplinary scientific expertise

Scientific advancement and an enabling environment to implement effective interventions are fundamental to the realisation of global targets for disease control. Alongside these factors, a strong political, social, and economic commitment is necessary at all levels of society to achieve success. Expertise from biomedical sciences, social sciences and economics, national programme delivery, patient/carer representatives, and civil society more broadly is necessary when setting new targets for disease control. National TB elimination programmes, for example, have only had sustained success in industrialised countries that saw rapid socioeconomic development and also addressed wider determinants of health. At the global level, the equitable engagement of political stakeholders is also crucial. For malaria, only three sub-Saharan African countries were present as full members at the WHA when the GMEP was adopted [34]. Global control strategies should, therefore, consult fully on target setting with national stakeholders from high-burden settings.

### Balance between ambition and caution when setting targets

A balance needs to be struck between ambitious, galvanising targets that drive funding, and ones that reflect the complexities and local epidemiological variations in disease. The 1991 leprosy elimination target was criticised for being arbitrary and ignoring the impact of secondary disabilities. However, subsequent targets have not driven new funding in the same way that this single target did. Global targets have been criticised for overlooking subpopulations of high incidence and promoting oversimplified, generalisable solutions to complex problems that are nuanced. On the other hand, numerous targets have been set for HIV and TB, many of which have not been met in high-burden populations but which have helped sustain funding commitments. There is a tension between time-limited elimination targets that can keep donor interest and the desire for community approaches that invest in social capital over a longer time but have the potential to be more equitable and better serve hard-to-reach groups [90]. While the language of eradication and elimination can prove seductive to donors, the risk exists of misdirecting efforts. Eradication requires significant up-front investment, which can cause its supporters to oversell its feasibility. With leprosy, achieving the global elimination target undoubtedly resulted in a decline in investment in research, even though pockets of high incidence and prevalence remain. For malaria, there is a risk of focusing elimination on areas of low incidence and prevalence to shrink the global malaria map, at the cost of control in high-incidence areas. There are therefore clear ethical considerations and consequences to be explored when setting ambitious goals and targets—particularly around their local effects and the opportunity costs of foregoing other areas of public health.

### Avoiding burdensome reporting and conflicting targets

In recent decades, the expansion of donor-driven disease programmes has led to a constant stream of so-called SMART (Specific, Measurable, Achievable, Relevant, Time-bound) targets and logframes (logical frameworks) to monitor projects and estimate impact [91]. There is a clear utility in employing these methods to develop intermediate and adaptable targets that allow for course corrections to programmes, and these can help address inequalities by including harder-to-reach subpopulations. However, a balance needs to be struck to avoid overly burdensome reporting requirements and the potential confusion of overlapping and sometimes conflicting targets both within and across vertical disease programmes. Linked to this is a clear need to consciously distinguish between process and outcome targets. As seen with HIV control, the achievement of process targets does not necessarily translate to a decrease in new infections or mortality.

### Retention of specialist skills

The experience with malaria and TB control in the 1970s and 1980s demonstrated a need to ensure retention of expert skills and specialist services while moving towards integrated health systems. The success of TB and HIV programmes are often intimately linked, with TB elimination in many settings dependent on adequate prevention and treatment of HIV [92]. Disease control programmes should therefore look for opportunities to jointly develop targets and integrate services and strategies where appropriate. The End TB strategy, for example, already predicates future achievement of its 2050 TB target on universal health coverage (UHC), social protection, and a range of SDG-related milestones, although relevant progress indicators have not been framed around these [71,93].

### Sustaining investment and political commitment as incidence falls

Finally, sustaining any elimination or eradication strategy in the end phase, as the cost per case averted increases, will require prolonged investment and continuing political buy-in. At present, 1.8 million people continue to be infected with HIV each year [17], and achieving elimination will only be possible with sufficient investment in research to develop vaccines, point-of-care diagnostics, and treatments; infrastructure upgrades that address wider determinants of health; and health and surveillance systems that allow for equitable delivery and access to services. For diseases like HIV that disproportionately affect marginalised groups, equity-based service delivery targets become even more important as the elimination strategy nears its end. Human rights-based approaches that explicitly seek to tackle stigma related to HIV and give key populations a stronger voice to influence policy will be vital to ensure that equitable progress is made to control disease across all sectors of affected populations [94].

## Supporting information

S1 Table Major global events or initiatives that influenced progress in disease control efforts. (DOCX)

## Abbreviations

ACT: artemisinin-based combination therapy
ART: antiretroviral therapy
ARV: antiretroviral
BCG: bacille Calmette-Guérin vaccine
DDT: dichlorodiphenyltrichloroethane
DOTS: Directly Observed Treatment, Short Course
G2D: grade 2 disability
GMEP: Global Malaria Eradication Campaign
HVTN: HIV Vaccine Trials Network
ITN: insecticide-treated bednet
LLIN: long-lasting insecticide-treated bednet
LMIC: low- or middle-income country
MDG: Millennium Development Goal
MDR TB: multidrug-resistant TB
MDT: multidrug therapy
PEPFAR: United States President’s Emergency Plan for AIDS Relief
PLHIV: person living with HIV/AIDS
RBM: Roll Back Malaria
SDG: Sustainable Development Goal
SMART: Specific, Measurable, Achievable, Relevant, Time-bound
TB: tuberculosis
UHC: universal health coverage
UNAIDS: Joint United Nations Programme on HIV/AIDS
UNGASS: UN General Assembly Special Session
USSR: Union of Soviet Socialist Republics
WHA: World Health Assembly
