# Supplementary material for: Setting targets for HIV/AIDS—What lessons can be learned from other disease control programmes?
Source: PLoS Med. 2019 Feb 4;16(2):e1002735. doi: 10.1371/journal.pmed.1002735 (PMC6361469; doi:10.1371/journal.pmed.1002735)
Supplement: S1 Table — (DOCX) [file pmed.1002735.s001.docx]

| **Malaria** | **Pre-1980s** | **1980s** | **1990s** | **2000s** | **2010s** |
| --- | --- | --- | --- | --- | --- |
| **Major policies and strategies** | - 1955 8th WHA approved GMEP - 1956 the WHO Expert Committee on malaria was called to design the eradication campaign - Focus on eradication using DDT to wipe out the mosquito vector and chloroquine to treat the disease, no investment in research of new technologies and disinvestment in control activities such as destruction of breeding marshes and prevention of mosquito bites - 1969, WHA recognise eradication in some countries is not feasible in the short term | - 1978 Alma Ata conference on primary health care - responsibility for malaria control activities shifts from technical experts to basic health care workers - 1982 launch of the Africa child survival initiative -combating childhood communicable diseases: control strategy relied on the use of antimalarial drugs in spite of the increasing resistance | - 1992 Global Malaria Control Strategy:   1) to provide early diagnosis and prompt treatment 2) plan and implement selective prevention methods 3) to detect, contain or prevent epidemics 4) to strengthen local capacities in basic and applied research   - Emphasis of the strategy is on a flexible, cost effective and sustainable programme adapted to local conditions - Action Plan for Malaria Control 1995 -2000: integration of malaria control activities into health delivery programmes | - 2008, RBM partnership the first Global Malaria Action Plan (GMAP) – for a malaria-free world 2008–2015 was endorsed by world leaders and the malaria community during the 2008 MDG Malaria Summit in New York. | - Launch of the WHO Global technical strategy for Malaria 2016-2030- “a malaria free world”, adopted by WHA in 2015 |
| **Key global events** | - 1940s the discovery of the first residual insecticide DDT - 1960s vector develops resistance to DDT and parasite develops resistance to chloroquine - Late 1960s USA involvement in Vietnam War drives investment into research for new antimalarial drugs - 1970s severe epidemics in several countries partly because of reduction in programme resources and a strong La Nina - 1970s economic crisis and countries supported the colonisation of their extensive primary forests by agriculture and mining leading to massive outbreaks. - Plus an intensive trade of antimalarial drugs, thus contributing to the spread of drug resistance | - Resurgence of malaria with disinvestment in malaria programmes, - Increasing resistance to chloroquine - A number of significant conflicts in West and Central Africa. - Impacts were greatest in rural areas where infrastructure was weakest | - 1990 WHA attributed the global resurgence of malaria to rapidly increasing antimalarial resistance, the lack of a clear control strategy, an acute shortage of financial resources. - 1992 World Declaration on the Control of malaria and a Global Malaria Control Strategy in Amsterdam, confirmed by WHA in 1993 - this was in response to a deteriorating situation. Strategy is outlined in the box above. - Harare Declaration on Malaria prevention and control in 1997 was designed to promote African economic recovery and development, but faltered due to the lack of support - 1997 Multilateral Initiative on malaria to encourage scientific research on malaria in Africa - 1998 Roll Back Malaria initiative conceived by WHO | - RBM initiative becomes RBM partnership involving WHO with UNDP, UNICEF and the World Bank - Abuja declaration in 2000 set objectives for RBM and got African political commitment - Oct 2007 Bill and Melinda Gates announce a new malaria eradication plan and this endorsed by Dr Margaret Chan, Director General of WHO at the same meeting | - Between 2000 and 2015 malaria incidence has decreased by 37% globally and mortality rates by 60% - Malaria Policy Advisory Committee to WHO set up and met for the first time in 2012 |
| **Funding** | - Late 1960's US invests again in malaria research programme for new antimalarials, because of involvement in Vietnam - 1963 US cuts its contribution to the WHO Malaria Special Account - 1970s UNICEF and other major collaborating agencies withdraw their support to malaria programmes in favour of general health programmes | - Funds for malaria control activities have dried up. DDT is banned in the US and 1982 production stops altogether. | - Global Malaria Control Strategy implementation was limited by human and financial resources - 1997 WHO DG allocated $10 million 24 African countries to accelerate implementation - Medicine for Malaria Venture – a non-profit foundation established in 1999 was funded by the World Bank, RBM and others – role in streamlining research on new antimalarial medicines | - Global Fund created in 2002 - World Bank’s Booster Programme - US President’s Malaria initiative set up 2005 - International funding for malaria rose from $149 million in 2000 to $1.66 billion in 2011. Majority from GFTAM |  |

**S1 Table: Major global events or initiatives that influenced progress**

| **Leprosy** | **Pre-1980s** | **1980s** | **1990s** | **2000s** | **2010s** |
| --- | --- | --- | --- | --- | --- |
| **Major policies and strategies** | Increasing dapsone resistance in the 1960s and availability of better bactericidal drugs (e.g. rifampicin) paved way for treatment of leprosy via a combination of drugs | **1981**: WHO Study Group on leprosy recommended fixed duration antibacterial multidrug therapy (dapsone and rifampicin for Paucibacillary leprosy; dapsone, rifampicin and clofazimine for Multibacillary leprosy) | In the early 1990s, the length of treatment with multidrug regimens (containing rifampicin) was reduced to a fixed period of 24 months. By the end of the 1990s, this was further reduced to a fixed period of only 12 months. | **2000-2005**: Final push to eliminate leprosy as a public health problem, focussing on multidrug therapy and passive case detection  **2006-2010**: Global strategy for further reducing the leprosy burden and sustaining leprosy control activities | **2011-2015**: Enhanced global strategy for further reducing the disease burden due to leprosy  **2016-2020**: Global Leprosy Strategy 2016‒2020. Three pillars are: Strengthen government ownership, coordination and partnership; Stop leprosy and its complications; Stop discrimination and promote inclusion |
| **Key global events** | **1966**: Ridley and Jopling propose their "classification of leprosy according to immunity: a five-group system". Treatment regimens in the 1980s would be assigned on the basis of this classification system  **1974**: WHO Special Programme for Research and Training in Tropical Diseases (TDR) established, with leprosy as one of their six core focus diseases  **1976**: THELEP (TDR Scientific Working Group on Chemotherapy of Leprosy) established to carry out field studies on dapsone resistance; clinical drug trials; development of new antileprosy drugs | **1984**: Pilot study launched to investigate feasibility of providing MDT as blister calendar packs. In 1987, Novartis introduced the first commercially available MDT blister pack, containing 4 weeks treatment  **1986**: Novartis Foundation for Sustainable development decides to directly support field programmes, to improve understanding of and to overcome barriers to accessing MDT | **1990s**: Growing divisions began to appear between WHO and ILEP regarding case definitions  **1995**: WHO begins providing free multidrug therapy to all endemic countries  **1999**: Inauguration of the Global Alliance for the Elimination of Leprosy (GAEL)  **1999**: MoU between WHO and Novartis/Novartis Foundation to freely provide sufficient quantities of high-quality MDT, in blister packs, to WHO for six years (2000–2005) to treat and cure all leprosy patients worldwide | **2000:** MDGs make no explicit mention of leprosy, or of disabled people or people affected by neglected tropical diseases  **2005**: *International Journal of Leprosy* ceases publication, noting absence of scientific evidence for the elimination policy  **2005**: WHO Kathmandu produced policy directives to reduce prevalence | **2012**: WHO roadmap on NTDs (covering 17 diseases, including leprosy)  **2013**: WHO and Nippon Foundation called an international Leprosy Summit to address "stagnation" in leprosy control. This resulted in the Bangkok Declaration, signed by health ministers of highly endemic countries, calling for renewed political commitment to leprosy control |
| **Funding** | **1974/5**: WHO accepts a $500,000 donation from Sasakawa for leprosy work, provided that an equivalent amount went to fill a funding shortfall in the Smallpox Eradication Programme  **1975**: Mr Ryochi Sasakawa provides a grant of US$ 502,000 to the WHO budget for leprosy, opening up opportunity to implement control programmes | **By 1980**: The Japanese Shipbuilding Industry Foundation (Ryoichi Sasakawa's foundation, later the Nippon Foundation) had provided $4m to WHO leprosy activities  In the early 1980s, ILEP provided annual contributions to LEP that ranged from US$400,000 to US$800,000  **1982**: JSIF made $600,000 available to WHO for implementing multidrug therapy.  **1983**: Damien Foundation of Belgium established drug fund for MDT with an initial endowment of US$400,000 | **1994**: At the First International Conference on Elimination of Leprosy, Sasakawa's Nippon Foundation announced that it would donate US$50 million to WHO over five years for MDT drugs  **1999**: At GAEL Launch, the Nippon Foundation pledged US$24 million over the period up to 2005, taking the total contribution of Nippon Foundation to WHO to nearly US$150 million | **2000**: Novartis begins donating drugs to patients through WHO | **2013:** Sasakawa Memorial Health Foundation committed to allocation increased funding for leprosy activities to support countries’ commitments made at the International Leprosy Summit  **2013:** Leprosy Research  Initiative launched to boost funding from outside ILEP organisations, and spends about €1.5 million each year on leprosy research |

| **TB** | **Pre-1980s** | **1980s** | **1990s** | **2000s** | **2010s** |
| --- | --- | --- | --- | --- | --- |
| **Major policies and strategies** | - Discovery of effective chemotherapy agents led to the building of vertical control programmes between 1948 - 1963 with mass case finding and specialised case management as the central technical strategy. - This proved too costly for LMICs which led to an integration of service delivery (1964-1976) into general health services and - Then an integration of managerial functions (1977-88) | - Integration of both TB service and managerial functions into general services and loss of specialist TB services and managers to increase reach and efficiency | - In 1993, WHO declared a "global tuberculosis emergency" - In 1994, WHO announced a new strategy focused on bacteriological detection among persons spontaneously presenting with symptoms to health service points and the provision of standardised short course chemotherapy, branded in 1995 as DOTS | - WHO and Stop TB partnership launched the first Global Plan to Stop TB 2001 -2005 - 2006 -2015 Stop TB strategy/Global Plan - explicitly addresses MDR TB and co-infection with HIV and TB while also promoting public-private mixed approaches and community engagement. - A fundamental change from the 1990’s DOTS strategy was the emphasis on patients centred care for all TB patients | - In 2014, 67th WHA approved the End TB strategy 2016 – 2035 - 2015 saw transition from MDGS to SDGs and Stop TB strategy to End TB strategy |
| **Key global events** | - In 1947 The first Expert Committee of WHO met in Paris and a TB section was established in the WHO secretariat - 1964 WHO Expert Committee on TB emphases the need to integrate service delivery into general health services now that TB treatment had been simplified and standardised - 1978 The WHO TB unit is transformed into a TB and respiratory infections unit | - 1985 saw the closure of UK Medical Research Council TB units - an example of the loss of interest in TB - In 1989, the WHO HQ staff for TB was two professionals managing a tiny budget | - In 1993 World Development Report emphasised that a sound strategy of tuberculosis control is one of the most cost effective health interventions available today. - In 1998 WHO convened an ad hoc meeting in London to discuss the global constraints to widening tuberculosis control and to identify potential solutions. - Stop TB initiative launched by WHO in 1998 | - 2000 Amsterdam ministerial conference to expand the global implementation of the DOTS strategy. - The United Nations Millennium Summit incorporated TB targets into the MDG - 2001 Formation of the Stop TB partnership - a platform to facilitate partnerships between health organisations, private companies, NGOs and research and funding institutions. - Civil Society mobilisation and more human approach to public health - 2001 Global Drug facility and Global TB Alliance - 2001 Green light committee enable access to affordable, high quality, second-line anti TB drugs for the treatment of MDR-TB. - 2009 WHA resolution on the prevention and control of MDR TB | - 2012 BDQ the first new TB drug in 40 years received FDA conditional approval - New Molecular diagnostics eg Xpert MTB/RIF or Genotype MTBDR plus assay are deployed to complex low resource settings - 16 new vaccine candidates |
| **Funding** |  |  |  | - 2002 Global Fund to fight AIDS, TB and Malaria founded - Global TB Drug Facility - 2006 UNITAID founded - Funding increased from $2.2 billion in 2002 to $8.3 billion in 2011 |  |

| **HIV** | **1980s** | **1990s** | **2000s** | **2010s** |
| --- | --- | --- | --- | --- |
| **Major policies and strategies** | **Late 1980s:** WHO began assisting countries to establish or strengthen National AIDS Programmes^[[1]](#endnote-1)^ | Shift away from viewing HIV/AIDS as global “pandemic” towards several different local epidemics with their own dynamics and epidemiological profiles  UN Development Programme sounded alarm of HIV/AIDS as a threat to development efforts in LMICs from early 1990s  Discovery of HAART and Brazil’s rights-based approach to providing free ART in 1996 helped transform HIV/AIDS from fatal to chronic illness | **2006**: Evidence that male circumcision could decrease the risk of female-to-male HIV transmission by 60% led WHO to recommend male circumcision in areas of high HIV prevalence and low male circumcision prevalence  **2007**: WHO issues new recommendations on HIV testing and counselling in health facilities, aiming to widen knowledge of HIV status and increase access to HIV treatment and prevention | **2011**: UNAIDS' Getting to Zero strategy (in tandem to the Political Declaration on HIV/AIDS) sets out the vision of the three zero's: "zero new HIV infections, zero AIDS-related deaths, and zero discrimination”  **2011:** Launch of the Global Plan towards the elimination of new HIV infections among children and keeping their mothers alive  **2014**: Moves towards the "fast-track" strategy, with a focus on rapid scale-up of essential HIV prevention and treatment approaches that will enable the response to outpace the epidemic  **2015**: WHO recommends "treat-all" policy, i.e. that anyone with HIV infection should begin antiretroviral treatment as soon as possible after diagnosis  **2016**: WHO publishes Global Health Sector Strategy on HIV 2016-2021 |
| **Key global events** | **1981**: First published reported cases of AIDS (though the term was not used until 1982)  **1985**: First serologic tests for HIV diagnosis  **1986:** Official naming of “human immunodeficiency virus” as cause of AIDS  **1987:** AZT approved to treat HIV  **1987**: WHO launched the Special Programme on AIDS, signalling the start of a global AIDS response  **1988:** WHO launched the Global Programme on AIDS | **1995:** WHO Office of HIV/AIDS and STD established  **1996**: Joint United Nations Programme on HIV/AIDS (UNAIDS) established  **1996**: HAART's effectiveness revealed at 11th International AIDS Conference in Vancouver | **2000**: Security Council meeting in January on HIV/AIDS, the first time the Security Council had discussed a health issue. Led to passing of Resolution 1308, stipulating that all peacekeeping operations should have an HIV prevention component  **2001**: UN General Assembly Special Session on HIV/AIDS - Declaration of Commitment on HIV/AIDS  **2001**: Doha Declaration on the TRIPS Agreement and Public Health, facilitating countries' generic manufacture of antiretrovirals  **2002**: South African government has been ordered by the country’s top legal body to provide all HIV-positive pregnant women and their newborn babies with the anti-AIDS drug nevirapine  **2002**: Establishment of Global Fund to fight HIV/AIDS, Tuberculosis and Malaria  **2003**: Establishment of President's Emergency Plan For AIDS Relief (PEPFAR)  **2006:** UNITAID founded to help with developing and widening access to ART and generic medicines. In **2010,** UNITAID created and invested in the Medicines Patent Pool (MPP) to negotiate voluntary licenses for HIV medicines. | **2011:** The HPTN 052 clinical trial showed that early initiation on ART in people living with HIV with a CD4 count between 350 and 550, reduced HIV transmission to HIV-negative partners by 96%. This demonstrated the feasibility of “treatment as prevention”  **2013:** Cuba becomes first certified country to eliminate mother to child transmission of HIV  **2015:** TB surpasses AIDS as the leading cause of deaths from infectious disease  **2016:** Warnings of complacency in the AIDS response issued at the 21^st^ International AIDS Conference in Durban |
| **Funding** | Annual funding for the AIDS response in LMICs went from $1 million in 1986 to $160 million by 1989 (UNAIDS estimates) | Annual funding for the AIDS response in LMICs surpassed $1 billion in the late 1990s, reaching an estimated $1.1 billion by 1999 | With additional resources mobilised by the Global Fund and PEPFAR, annual funding for the AIDS response in LMICs increased massively to reach $15.9 billion by 2009 | At the end of 2016, US$ 19.1 billion was invested in the AIDS response in LMICs  Domestic resources constituted 57% of the total resources for HIV in low- and middle-income countries in 2015  Donor funding for the AIDS response is in decline; analysis by the Kaiser Family Foundation found that donor government spending to address HIV LMICs declined by more than $1 billion between 2014 and 2015 (US$7.53 billion in 2015 compared to US$8.62 billion in 2014), a 13% decline^[[2]](#endnote-2)^ |

1. World Health Organisation. *Global Programme on AIDS 1987-1995: Final Report with Emphasis on the 1994-1995 Biennium.* 1997. Available from: <http://apps.who.int/iris/bitstream/10665/65955/1/WHO_ASD_97.1.pdf> [Accessed 19th June 2017] [↑](#endnote-ref-1)
2. Kaiser Family Foundation. *Financing the Response to HIV in Low- and Middle-Income Countries: International Assistance from Donor Governments in 2015.* 2016. Available from: <http://files.kff.org/attachment/Financing-the-Response-to-HIV-in-Low-and-Middle-Income-Countries-International-Assistance-from-Donor-Governments-in-2015> [Accessed 19th June 2017] [↑](#endnote-ref-2)
